# Association of Mitochondrial Variants with the Joint Occurrence of Polycystic Ovary Syndrome and Hashimoto’s Thyroiditis

**DOI:** 10.3390/antiox12111983

**Published:** 2023-11-08

**Authors:** Natalia Zeber-Lubecka, Maria Kulecka, Katarzyna Suchta, Michalina Dąbrowska, Michał Ciebiera, Ewa E. Hennig

**Affiliations:** 1 Department of Gastroenterology, Hepatology and Clinical Oncology, Centre of Postgraduate Medical Education, 02-781 Warsaw, Poland; nzeber@cmkp.edu.pl (N.Z.-L.); mkulecka@cmkp.edu.pl (M.K.); 2 Department of Genetics, Maria Sklodowska-Curie National Research Institute of Oncology, 02-781 Warsaw, Poland; michalina.dabrowska@nio.gov.pl; 3 Department of Gynaecological Endocrinology, Medical University of Warsaw, 00-315 Warsaw, Poland; katarzyna.suchta@wum.edu.pl; 4 Second Department of Obstetrics and Gynecology, Centre of Postgraduate Medical Education, 00-189 Warsaw, Poland; mciebiera@cmkp.edu.pl; 5 Warsaw Institute of Women’s Health, 00-189 Warsaw, Poland

**Keywords:** polycystic ovary syndrome, Hashimoto’s thyroiditis, coexisting PCOS and HT, mitochondrial variants, oxidative stress

## Abstract

Background. The prevalence of Hashimoto’s thyroiditis (HT) among women with polycystic ovary syndrome (PCOS) is higher than in the general female population, but the factors predisposing to the coexistence of these disorders remain unclear. This study employed whole genome sequencing of mitochondrial DNA to identify genetic variants potentially associated with the development of PCOS and HT and predisposing to their joint occurrence. Results. A total of 84 women participated, including patients with PCOS, HT, coexisting PCOS and HT (PCOS + HT) and healthy women. Both Fisher’s exact and Mann–Whitney U statistical analyses were performed to compare the frequency of variants between groups. Ten differentiating variants were common to both analyses in PCOS + HT vs. PCOS, one in PCOS + HT vs. HT, and six in PCOS + HT vs. control. Several variants differentiating the PCOS + HT group from PCOS and controls were identified, located both in the mitochondrial genes (including the *MT-CYB*, *MT-ND1*, *MT-ND2*, *MT-ND4*, *MT-ND6*, *MT-CO1*, *MT-CO3*) and the D-loop region. Only two variants differentiated PCOS + HT and HT groups. One variant (13237a in *MT-ND5*) was common for all three comparisons and underrepresented in the PCOS + HT group. Functional enrichment analysis showed 10 pathways that were unique for the comparison of PCOS + HT and PCOS groups, especially related to ATP production and oxidative phosphorylation, and one pathway, the NADH-quinone oxidoreductase, chain M/4, that was unique for the comparison of PCOS + HT and control groups. Notably, nine pathways shared commonality between PCOS + HT vs. PCOS and PCOS + HT vs. control, related to the biogenesis and assembly of Complex I. Conclusion. This study provides novel insights into the genetic variants associated with oxidative stress in women with coexisting PCOS and HT. Mitochondrial dysfunction and oxidative stress appear to play a role in the pathogenesis of both conditions. However, more mitochondrial variants were found to differentiate women with both PCOS and HT from those with PCOS alone than from those with HT alone.

## 1. Introduction

Polycystic ovary syndrome (PCOS) and Hashimoto’s thyroiditis (HT) belong to the most common endocrine disorders diagnosed in women of reproductive age and are often undiagnosed for a long time [1]. These two conditions share numerous similarities. Several studies indicated that HT and increased levels of antithyroid antibodies were more common in women with PCOS than in the general female population (22–28% vs. 8–10%) [1]. The metabolic and reproductive consequences of the disease occur more frequently and are more severe in women with coexisting PCOS and HT than in women with each condition separately [2,3]. Genetic factors were shown to play a significant role in the etiology of both diseases [1]. However, a potentially predisposing factor that contributes to this comorbidity is still unknown.

Mitochondria play a fundamental role in cell energy metabolism, apoptosis and proliferation [4,5]. These organelles contain an independent multi-copy genome, and are the main endogenous source of reactive oxygen species (ROS) [6,7]. At appropriate levels, ROS have a key influence on the biological functions of mammalian cells, including cell division, metabolism, gene expression, and immune response [7]. Mutations in mitochondrial DNA (mtDNA) may result in respiratory chain dysfunction, leading to reduced ATP production and the overproduction of ROS, and are involved in the development of various diseases [5,8,9]. Increased oxidative stress (OS) and decreased antioxidant levels are correlated with the progression of PCOS and diverse phenotypic features of the disease, including obesity, insulin resistance (IR) and hyperandrogenism [10,11].

Several variants in mitochondrial genes encoding transfer RNA (mt-tRNA), 12S and 16S ribosomal RNA (mt-rRNA) were identified in women with PCOS [12]. Mutations affecting highly conserved nucleotides in mitochondrially encoded tRNA serine 1 [tRNASer(UCN); *MT-TS1*] and NADH-ubiquinone oxidoreductase chain 5 protein (*MT-ND5*), critical for their stability and biochemical function, may lead to mitochondrial dysfunction and are likely to be involved in the pathogenesis of PCOS [13]. Additionally, mitochondrial copy number (CN) analysis suggested that mitochondrial dysfunction might be involved in the pathogenesis of IR in PCOS women as well as represent an independent risk factor for PCOS [14,15,16]. However, it should be noted that the majority of those studies were conducted in Asian populations. One study indicated significantly higher mitochondrial DNA CN and mitochondrial oxidative damage in HT patients [17]. To date, mtDNA variations have not been investigated in terms of PCOS and HT comorbidity.

Emerging evidence suggests a potential link between genetic factors and alterations in mitochondrial function, including OS, in the pathogenesis of PCOS and HT. In this study, whole genome sequencing (WGS) of mtDNA was used to identify mtDNA genetic variants potentially associated with the development of PCOS and HT and predisposing to the joint occurrence of these conditions.

## 2. Materials and Methods

### 2.1. Ethics Statement

The local ethics committee approved the study (Medical University of Warsaw, No: KB/200/2015 and Centre of Postgraduate Medical Education, No: 77/PB/2017, Warsaw, Poland). All enrolled patients and control subjects were Polish Caucasians and provided written informed consent before they participated in the study. The study protocol conformed to the ethical guidelines of the 1975 Declaration of Helsinki.

### 2.2. Study Population

This case–control study included 84 women, 20 in each study group: PCOS, HT, PCOS coexisting with HT (PCOS + HT), and 24 healthy women in the control group. All participants were of European ancestry. Strict inclusion and exclusion criteria were used to recruit the patients. In regard to the diagnosis of PCOS, the patients had to meet all three so-called Rotterdam criteria [18], i.e., chronic anovulation or infrequent ovulation, hyperandrogenism manifesting by elevated androgen levels, and the presence of polycystic ovarian morphology on ultrasound examination. The inclusion criteria for patients with HT included an elevated serum level of anti-thyroid peroxidase (TPO) and anti-thyroglobulin (Tg) antibodies, and hypoechogenicity of the thyroid gland on the ultrasound image. Due to additionally diagnosed clinical or subclinical hypothyroidism, 50% of HT patients in both HT and PCOS + HT groups received levothyroxine. At the time of blood collection, all patients with HT were euthyroid, either because hypothyroidism had not yet developed or as a result of adequate supplementation.

The exclusion criteria were: refusal to participate in the study and the diagnosis of hyperandrogenism due to causes other than PCOS, such as non-classic adrenal hyperplasia, androgen-secreting tumors, or Cushing syndrome. The use of drugs, supplements or herbs that could affect the hormonal function and serum androgen levels, for up to six months before participating in the study was an additional exclusion criterion. Baseline serum levels of relevant autoantibodies, hormones, and endocrine parameters were determined as part of routine diagnostic procedures. The clinical characteristics, hormonal, and biochemical features of the enrolled patients and control subjects are shown in Table 1.

### 2.3. Nucleic Acid Extraction

Total genomic DNA was isolated from whole blood samples treated with EDTA using QIAamp DNA Mini Blood Kit (Qiagen GmbH, Hilden, Germany) following the manufacturer’s instructions. The quantity and purity of extracted DNA were measured using Qubit™ dsDNA HS Assay Kit on Qubit fluorometer 2.0 (Thermo Fisher Scientific, Waltham, MA, USA) and NanoDrop 2000 spectrophotometer (Thermo Fisher Scientific, Waltham, MA, USA), respectively.

### 2.4. Mitochondrial DNA Sequencing and Variant Calling

MtDNA libraries were prepared using the Precision ID mtDNA Whole Genome Panel, a multiplex assay with two primer pools aimed at analyzing the 16,569 bp human mitochondrial genome. The mtDNA libraries were sequenced on the Ion Torrent platform (Thermo Fisher Scientific, Waltham, MA, USA) using Ion PI™ Hi-Q™ Sequencing Kit reagents. Mitochondrial variants and haplogroups were identified and assessed with HID Genotyper Ion torrent Plugin (v2.1_df70f6b). Haplogroup assignment to the higher level was based on the hierarchization of the phylogenetic tree (http://www.phylotree.org/, accessed on 1 May 2022). Only variants described as True Variants or Heteroplasmies were considered for a downstream analysis. Length heteroplasmies were analyzed only if they were marked as present in the European DNA profiling group (EDNAP) mtDNA population database (EMPOP). The variants were annotated according to extended guidelines for mitochondrial variant typing [19], with small letters denoting deletions, and capital letters accompanied by a number, such as 309.1C, denoting insertions. Deletions with allele frequency higher than 50% were simply denoted with the “del” symbol instead. The International Union of Pure and Applied Chemistry (IUPAC) ambiguity codes were used for single-nucleotide heteroplasmies.

### 2.5. Statistical Analyses

We conducted two types of statistical analyses, the Fisher’s exact test and the Mann–Whitney U test, in order to compare the occurrence of mitochondrial variants across different groups. Differences in variant presence/absence between groups were compared using the Fisher’s exact test (implemented in base R). Allele frequencies were compared with the Mann–Whitney U test. The *p*-value was adjusted (*p_adj_*-values) for multiple comparisons with the Benjamini–Hochberg (B-H) algorithm [20], assuming *p_adj_*-value < 0.05 as the significance threshold. Functional enrichment analysis for selected genes was conducted using the Cytoscape platform v.3.6.1 with the ClueGO v.2.5.1 application [21] based on the Reactome Pathway and the Kyoto Encyclopedia of Genes and Genomes (KEGG) databases, with *p_adj_*-value < 0.05 as the threshold level. Biochemical parameters were compared with the Kruskal–Wallis test, with the Mann–Whitney U test as a post hoc test, and control group as a reference. Variant significance assessment was performed with mvTool version 7.1 [22]. Used databases included: HmtDB [23] and ClinVar [24]. The following tools were used to predict pathogenicity in silico: CADD [25] and MitoTip [26]. The interpretation for MitoTip score was provided as a result. For CADD, a score above 10 is considered pathogenic (following the interpretation of Kelsen et al. [27]).

## 3. Results

### 3.1. Identified mtDNA Haplogroups

A total of 70 distinct mtDNA subhaplogroups were identified in 84 participating women. A group of 23 women were characterized into nine subhaplogroups, including H, H13a1a1b, H1a3, J1b1a1, K1a4a1d, K1b2a2, T2b, U4a1 and W. Seven out of 23 women were classified as H subhaplogroup. The remaining 61 women were characterized by unique subhaplogroups (Supplementary Table S1). After the hierarchization of the phylogenetic tree, nearly all patients were assigned to three main haplogroups, R0, U and JT (Table 2). As many as 50% of HT patients were classified as haplogroup R0, while haplogroup U dominated among women with PCOS, tested in 40% of this group. The JT haplogroup was by far the least abundant, although there was a noticeable increasing trend in the control group. Although the obtained results were not statistically significant, they indicated that the study groups were homogeneous and included women belonging to the Central European population.

### 3.2. Significantly Differentiating Mitochondrial Variants 

Two kinds of statistical analyses were performed, the Fisher’s exact and Mann–Whitney U tests, to compare the frequencies of mitochondrial variants between groups. Fisher’s exact test was used to assess disparities in the presence or absence of variants (significant over- or underrepresentation of variants) among the groups, while the Mann–Whitney U test was employed to compare allele frequencies (differences in the percentage share of variants). As shown in Table 3, pairwise comparison analyses between PCOS + HT group and the other three groups (PCOS or HT alone and controls) identified several mitochondrial variants with significant (*p* < 0.05) frequency differences in at least one analysis. Of them, 3 and 23 variants significantly differentiated the PCOS + HT group in all three comparisons after *p*-value adjustment, in the Fisher’s exact and Mann–Whitney U analyses, respectively (Table 3).

**Figure 1:**
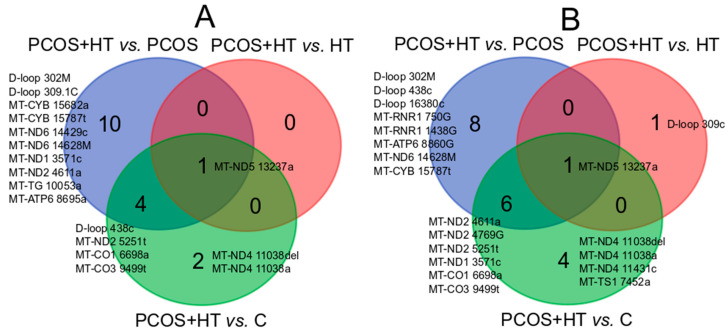
Significantly differentiating variants and assigned genes in comparisons of PCOS + HT and PCOS, HT and control (C) groups; (**A**)—Fisher’s exact analysis; (**B**)—Mann–Whitney U analysis.

As presented in a Venn diagram (Figure 1A), Fisher’s analysis revealed 14 variants unique for the comparisons of the PCOS + HT group with the PCOS and control (C) groups. All of them were underrepresented in patients with coexisting PCOS and HT, including two variants located in the *MT-ND6* (14628M, 14429c) gene, two in *MT-ND2* (4611a, 5251t), two in *MT-CYB* (15787t, 15682a) and two in the D-loop region (302M, 309.1C) (Table 3). Additional two variants located in the *MT-ND4* gene were unique for PCOS + HT vs. C, while 11038del was underrepresented and 11038a was overrepresented in the PCOS + HT group. The 13237a variant in the *MT-ND5* gene was common for all three comparisons and was underrepresented in the PCOS + HT group. No unique variant was shown for the comparison between PCOS + HT and HT alone.

The Mann–Whitney U test was effective in detecting slightly more significantly differentiating variants compared to the Fisher’s test (Table 3). Ten, one and six variants were common for both methods of analysis, in the comparisons of the PCOS + HT group and the PCOS, HT and control groups, respectively. Two new variants, located in *MT-TS1* (7452a) and *MT-ND4* (11431c), were unique for PCOS + HT vs. C (*p_adj_* < 0.05), and the 309c variant located in the D-loop region was unique for PCOS + HT vs. HT (*p* < 0.05) (Figure 1B). Interestingly, five variants significantly differentiating the PCOS + HT and PCOS groups only in the Mann–Whitney U analysis, including 8860G (*MT-ATP6*), 4769G (*MT-ND2*), 1438G and 750G (*MT-RNR1*) and 16380c in the D-loop region, belong to ancestral mitochondrial variants (Table 3). Further, selected variants’ significance was assessed and summarized in Supplementary Table S4. Based on ClinVar and HmtDB MitoTip databases, variants 15682a (*MT-CYB*) and 14628M (*MT-ND6*) differentiating in PCOS + HT vs. PCOS were classified as probable pathogenic and pathogenic, respectively.

### 3.3. Functional Enrichment Analyses

Functional enrichment analysis was performed for genes assigned to the selected significantly differentiating variants according to Fisher’s exact test. We identified 19 and 10 biological processes or signaling pathways enriched by variants differentiating in PCOS + HT vs. PCOS and PCOS + HT vs. C groups, based on the Reactome Pathway and the KEGG databases, respectively (*p_adj_* < 0.05) (Supplementary Tables S2 and S3).

**Figure 2:**
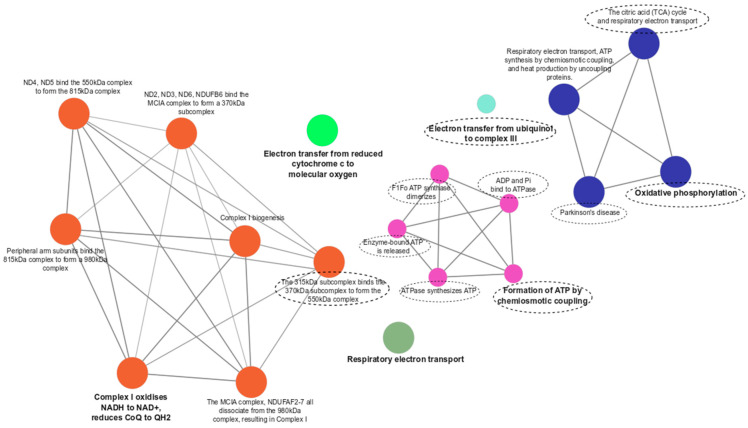
Functional enrichment analysis for variants differentiating PCOS + HT and PCOS groups. Bold indicates the most significant functional pathways that define the name of the signaling pathway group (*p*-value adjusted with the Benjamini–Hochberg). Unique pathways marked with a dashed line.

**Figure 3:**
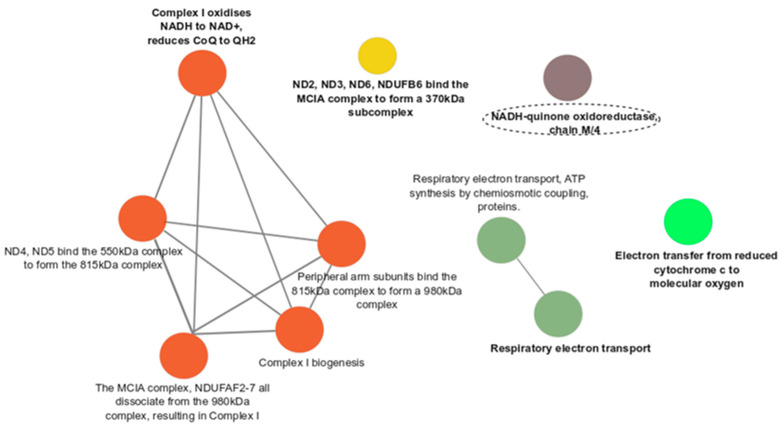
Functional enrichment analysis for variants differentiating PCOS + HT and control groups. Bold indicates the most significant functional pathways that define the name of the signaling pathway group (*p*-value adjusted with the Benjamini–Hochberg). Unique pathways are marked with a dashed line.

Functional enrichment analysis showed 10 pathways that were unique for the comparison of PCOS + HT and PCOS groups, especially related to ATP production and oxidative phosphorylation (OXPHOS), including the ADP and Pi bind to ATPase, ATPase synthesizes ATP, Electron transfer from ubiquinol to cytochrome c of complex III, Enzyme-bound ATP is released, F1Fo ATP synthase dimerizes, Formation of ATP by chemiosmotic coupling, Oxidative phosphorylation, Parkinson’s disease, The 315 kDa subcomplex binds the 370 kDa subcomplex to form the 550 kDa complex, and The citric acid (TCA) cycle and respiratory electron transport (Figure 2). In turn, only one unique pathway, the NADH-quinone oxidoreductase, chain M/4, was found for the PCOS + HT and control group comparison (Figure 3).

Another nine pathways, mainly related to the Complex I (CI) assembly pathway, were common for PCOS + HT vs. PCOS and PCOS + HT vs. C, including the Complex I oxidises NADH to NAD+, reduces CoQ to QH2; Complex I biogenesis; Respiratory electron transport; Electron transfer from reduced cytochrome c to molecular oxygen; ND2, ND3, ND6, NDUFB6 bind the MCIA complex to form a 370 kDa subcomplex; ND4, ND5 bind the 550 kDa complex to form the 815 kDa complex; Peripheral arm subunits bind the 815 kDa complex to form a 980 kDa complex; Respiratory electron transport, ATP synthesis by chemiosmotic coupling, and heat production by uncoupling proteins; The MCIA complex, NDUFAF2-7 all dissociate from the 980 kDa complex, resulting in Complex I.

Functional enrichment analysis showed no pathway significantly enriched by genes assigned to variants differentiating in PCOS + HT vs. HT, suggesting that patients with the joint occurrence of PCOS and HT and HT alone share similar mitochondrial variants.

## 4. Discussion

The incidence of thyroid dysfunction in a group of women with PCOS is about three times higher than that in healthy women [1]. However, no conclusive explanation for this phenomenon can be provided. By applying the mitochondrial WGS strategy, the current study highlights novel mtDNA genetic variants associated with the OS in patients with PCOS and HT and possibly predisposing to the joint occurrence of these conditions in the Polish population.

In this study, Fisher’s exact and Mann–Whitney U association analyses revealed several new unique variants in mitochondrial genes and the D-loop non-coding sequence, differentiating the group of patients with coexisting PCOS and HT from groups of patients with PCOS or HT alone and the controls (Figure 1). One variant, 13237a, located in the *MT-ND5* gene was common for all three comparisons, suggesting that it may be unique to PCOS + HT patients. The identified variants were mostly underrepresented in the PCOS + HT group (Table 3). We identified variant 15682a (*MT-CYB*) differentiating in comparison PCOS + HT vs. PCOS clinically classified as probable pathogenic in familial breast cancer [28].

To date, six variants in mitochondrial tRNA genes, including tRNAGln (*MT-TQ*), tRNACys (*MT-TC*), tRNAAsp (*MT-TD*), tRNALys (*MT-TK*), tRNAArg (*MT-TR*) and tRNAGlu (*MT-TE*), seven variants in the 12S rRNA gene and three variants in 16S rRNA have been described as being associated with PCOS in the Chinese population [12]. The authors suggested that mutations in mtDNA, especially the OXPHOS complex and tRNAs, might be associated with PCOS development. Also, Reddy et al. [29] showed a significant association of D310 and A189G variants in the mtDNA D-loop region, non-coding and much more variable sequence, and the reduction in mtDNA CN in patients with PCOS compared to control individuals. In turn, Ding et al. [16] identified nine mt-tRNA mutations that were associated with PCOS-IR: A3302G and C3275A in tRNALeu(UUR) (*MT-TL1*), T4363C and T4395C in tRNAGln (*MT-TQ*), C7492T in tRNASer(UCN) (*MT-TS1*), A7543G in tRNAAsp (*MT-TD*), A8343G in tRNALys (*MT-TK*), T10454C in tRNAArg (*MT-TR*) and A14693G in tRNAGlu (*MT-TE*).

Several wide-scale studies linked HT to the m.3243A > G mutation in the tRNALeu (UUR) gene (*MT-TL1*), the most observed pathogenic mtDNA mutation, which has a high phenotypic variability involving different organs such as the brain and nerves, the skeletal muscle, heart, the endocrine system, the gastrointestinal tract, and the skin [30]. Some phenotypes correspond to well-known syndromes such as mitochondrial encephalomyopathy syndrome (MELAS) or Leigh syndrome. Chin et al. [31] conducted an observational study in 35 patients with this mutation and showed that the incidence of endocrine-related medical issues included 51.4% of cases with diabetes, 22.9% with low lactate levels, 8.6% with elevated pyruvate levels and 5.7% of cases with hypothyroidism. In addition, Muller-Hocker et al. [32] reported that a common deletion of 4977 bp (position 8482 to 13,459) in the mtDNA of thyroid oxyphil cells of 61-year-old woman with HT was linked to a respiratory chain dysfunction.

To our knowledge, there were no studies using wide-scale methods to explore the impact of mitochondrial variants on HT development. However, it should be considered that the limitation of this research was the relatively small sample size of each group. Our Mann–Whitney U analysis showed only one variant (309.1c) in the D-loop region differentiating women with coexisting PCOS and HT from those with HT alone (Table 3). Although our knowledge in this field is still scant, patients with both HT and PCOS and HT alone seem to share similar mitochondrial variants.

Oxidative phosphorylation and the citric acid (TCA) cycle were among the unique pathways enriched by genes assigned to variants differentiating PCOS + HT and PCOS groups, including *MT-ATP6, MT-COX1, MT-COX3, MT-CYTB, MT-ND1, MT-ND2, MT-ND5, MT-ND6* (Figure 2, Supplementary Table S2). Mitochondria belong to the key players in generating and regulating cellular bioenergetics, producing the majority of OXPHOS molecules [33]. It was shown that women with PCOS might exhibit impaired OXPHOS efficiency and increased OS [10,34]. OS contributes to IR, which is a common feature of PCOS [35]. Elevated insulin levels stimulate the ovaries to produce excessive androgen hormones, including testosterone, which disrupts normal ovulation cycle and contributes to the development of PCOS symptoms [36]. Furthermore, impaired mitochondrial function and OS within the ovarian follicles may lead to altered energy metabolism, reduced egg quality, and compromised reproductive outcomes [37,38]. Similarly to PCOS, impaired OXPHOS has several consequences for HT development [39]. Decreased energy production affects the overall function of the thyroid gland. Additionally, mitochondrial dysfunction may contribute to increased inflammation, cell damage, and apoptosis in the thyroid tissue, exacerbating the autoimmune response [40,41]. Thyroid hormones themselves play a role in regulating mitochondrial function and OXPHOS [42]. Alterations in thyroid hormone levels in HT impact mitochondrial function and energy metabolism [43].

The TCA cycle is an integral part of cellular respiration. It occurs in the mitochondria and is responsible for the oxidation of acetyl-CoA, derived from carbohydrates, fats, and proteins, into carbon dioxide and the generation of reducing equivalents, NADH, and FADH2. These reducing equivalents are critical for the subsequent OXPHOS process in the electron transport chain, leading to the production of ATP. The oocyte obtains the required energy to complete the maturation process from the cumulus–oocyte complex due to, among others, the TCA cycle. The disruption of citrate formation in this process indicates reduced oocyte competence [44,45]. In mice with dehydroepiandrosterone (DHEA)-induced hyperandrogenism, oocytes exhibited lower citrate levels, glucose-6-phosphate dehydrogenase (G6PD) activity and lipid content, suggesting abnormal TCA cycle metabolism and the pentose phosphate pathway [46]. Thus, elevated DHEA levels in women with hyperandrogenism and PCOS may have a negative impact on oocyte function and contribute to impaired pregnancy. In addition, the hepatic pathophysiology of PCOS [47,48] was linked to the downregulation of the TCA cycle and glycolysis processes, regardless of age, based on the hepatic exosome metabolome analysis in a mouse model [49].

In turn, thyroid hormones promote the entry of glucose and fatty acids into cells, increasing the supply of substrates for cellular respiration, including the TCA cycle [50]. In addition, these molecules boost the activity of enzymes involved in the TCA cycle and the electron transport chain, leading to increased ATP production [51]. In hypothyroidism, with reduced levels of thyroid hormones, the overall cellular metabolism tends to slow down. This may impact the TCA cycle and its associated processes, leading to reduced ATP production and cellular energy levels [52]. Consequently, individuals with hypothyroidism may experience symptoms such as fatigue, weight gain, cold intolerance, and a slower heart rate.

Decreased OXPHOS activity is associated with the deficiency of NADH-quinone oxidoreductase (CI), the first and the largest complex of the OXPHOS system [53]. In our study, the NADH-quinone oxidoreductase, a chain M/4 pathway, was the only unique pathway when comparing a group of patients with coexisting PCOS and HT and a control group of healthy women (Figure 3). Abnormal CI function was associated with the shape of mitochondrial structures and increased levels of ROS [53], possibly leading to mitochondrial fragmentation where the cellular antioxidant defense systems were not properly balanced.

Metformin is a widely accepted medication for treating metabolic consequences of PCOS [54]. However, its mechanism of action remains unclear. It appears to specifically reduce oxygen flow through substrates for CI [55], suggesting that its androgen-lowering effect is mediated by the specific inhibition of CI of the mitochondrial respiratory chain. Thus, the direct inhibition of CI by other specific agents should result in reduced androgen production [55].

CI transfers electrons from NADH to coenzyme Q, contributing to the balance of cellular redox state [56]. At normal levels (the euthyroid state), thyroid hormones enhance various metabolic processes, including those involved in the electron transport chain, leading to the increased activity of CI and other electron transport complexes, and higher ATP production through OXPHOS. The disruption of this process due to hypothyroidism may result in decreased CI activity and ATP production, leading to altered cellular redox balance and potentially increased OS [56,57].

The respiratory electron transport, ATP synthesis by chemiosmotic coupling, and heat production by uncoupling proteins pathway, the common pathway for comparisons of the PCOS + HT group with both PCOS and control groups, was represented by variants in the *MT-ATP6, MT-COX1, MT-COX3, MT-CYTB, MT-ND1, MT-ND2, MT-ND5, MT-ND6 and MT-COX1, MT-COX3, MT-ND2, MT-ND4, MT-ND5* genes, respectively (Supplementary Table S2). Mitochondrial uncoupling protein 1 (UCP1) is a key component of heat production in brown adipose tissue (BAT) [58]. This mechanism was observed in women with PCOS exhibiting reduced BAT function, resulting from high androgen levels [59]. In rats with DHEA-induced PCOS, the reduced expression of thermogenic genes, UCP1 and mitochondrial OXPHOS proteins was observed, which affected the defect in energy metabolism and BAT activity [60]. In turn, thyroid hormones were identified as major modulators of energy production and thermogenesis, promoting proton leakage and increased OS, in which UCP1 is involved [42].

In this study, the mtDNA haplogroups of women with PCOS, HT and the joint occurrence of both disorders in the Polish population were determined for the first time. Given the lack of data, these results represent a new point around genetic variant search in relation to ancestry. So far, the relationship between classification into specific haplogroups and the pathophysiology of PCOS and HT has not been determined [61]. Based on the detected mitochondrial variants, all women participating in the study were classified into specific haplogroups. The most frequently observed ones were R0, U and JT (Table 2). This result, while statistically insignificant, stays in line with the dominant haplogroups observed in the European population [62]. However, in regard to the whole Slavic population, HV, U and JT were the dominant described haplogroups, occurring in 90% of the population [63].

## 5. Conclusions

This is the first study in which mitochondrial variant analysis was performed in a population of women with the joint occurrence of PCOS and HT. Both of these disorders are complex conditions influenced by numerous factors, and OS appears to be involved in their pathogenesis. However, the exact relationship between OS and these conditions is not fully understood. Therefore, further research is needed to elucidate the underlying mechanisms. It should be noted that OS is most likely not the sole cause of PCOS or HT, but rather one of contributing factors including genetic predisposition, epigenetic modifications, and environmental influences. While our findings provide valuable insights into the topic at hand, further validation through external, independent datasets or studies is necessary to confirm the generalizability of our results. It is important for future studies to replicate our findings in larger and more diverse patient populations to enhance the robustness of the results.

## Figures and Tables

**Table 1:** Clinical and hormonal parameters of study participants.

**Parameter**	**PCOS** ***N* = 20**	**HT** ***N* = 20**	**PCOS + HT** ***N* = 20**	**Control** ***N* = 24**	**Kruskal–Wallis**
	**Median (IQR)**	**Median (IQR)**	**Median (IQR)**	**Median (IQR)**	***p_adj_*-Value**
BMI (kg/m^2^)	23.52 (7.75)	**22.11 (3.99)**	26.77 (10.16)	**21.63 (2.85)**	ns.
Age (years)	24 (8)	**33.5 (5.75)**	25 (3.25)	**30 (8)**	7.9 × 10^−4^
FSH (mIU/mL)	5.19 (1.93)	5.19 (2.12)	4.43 (1.02)	**5.55 (1.07)**	ns.
LH (mIU/mL)	8.66 (5.52)	5.3 (2.79)	6.59 (4.15)	4.77 (0.85)	1.9 × 10^−3^
LH/FSH	1.7 (0.97)	1.01 (0.79)	1.33 (1.03)	**0.87 (0.23)**	5.3 × 10^−4^
E_2_ (pg/mL)	36.5 (18)	39 (37.75)	41.5 (12)	32.5 (20.25)	ns.
T (ng/mL)	0.55 (0.16)	**0.32 (0.12)**	0.53 (0.17)	**0.32 (0.12)**	8.9 × 10^−9^
A (ng/mL)	4.46 (1.63)	**1.94 (0.59)**	3.77 (1.95)	**1.96 (0.23)**	6.6 × 10^−11^
PRG (ng/mL)	0.3 (0.2)	**9.75 (9.12)**	0.27 (0.44)	**11 (4.38)**	1.9 × 10^−9^
17-OH-PRG (ng/mL)	2.65 (0.97)	1.27 (0.85)	1.42 (1.16)	1.23 (0.45)	5.1 × 10^−3^
DHEAS (µmol/l)	9.24 (3.52)	**6.28 (4.3)**	9.51 (5.45)	**6.88 (2.98)**	1.5 × 10^−2^
TSH (µIU/mL)	1.31 (0.77)	1.31 (1.15)	1.66 (1.07)	1.57 (0.8)	ns.
fT_4_ (pmol/mL)	**12.43 (1.46)**	13.23 (1.84)	13.74 (1.9)	**12.5 (0.64)**	1.7 × 10^−2^
TPO-Ab (IU/mL)	**0.3 (0.2)**	276.5 (601)	122 (219.25)	**0.69 (0.64)**	6.6 × 10^−11^
Tg-Ab (IU/mL)	**1.75 (1.79)**	41.5 (58.25)	34.5 (35.5)	**1 (0.51)**	6.6 × 10^−11^

Data are shown as the median and interquartile range (IQR). The bold value indicates statistically significant differences revealed in comparison with PCOS + HT group (*p* < 0.05). A, androstenedione; BMI, body mass index; DHEAS, dehydroepiandrosterone sulfate; E2, estradiol; FSH, follicle-stimulating hormone; fT4, free thyroxine; LH, luteinizing hormone; PRG, progesterone; T, testosterone; Tg-Ab, anti-thyroglobulin antibody; TPO-Ab, anti-thyroid peroxidase antibody; TSH, thyroid-stimulating hormone. ns., not significant.

**Table 2:** The main mtDNA haplogroups detected after the hierarchization of the phylogenetic tree.

Haplogroup	PCOS % in Group *N* = 20	HT % in Group *N* = 20	PCOS + HT % in Group *N* = 20	Control % in Group *N* = 24
R0	35	50	35	33
U	40	30	30	29
JT	10	10	20	25
other	15	10	15	13

**Table 3:** Significantly differentiating variants revealed by Fisher’s exact and Mann–Whitney U analyses.

Fisher’s Exact Test					Mann–Whitney U Test			
PCOS + HT vs. PCOS								
Gene	Variant	Estimate	*p*-Value	*p_adj_*-Value	Gene	Variant	*p*-Value	*p_adj_*-Value
*MT-CO1*	6698a	0	1.29 × 10^−5^	**0.0049**	*MT-ATP6*	8860G	4.72 × 10^−6^	**0.0002**
*MT-CO3*	9499t	0	4.51 × 10^−5^	**0.0085**	*MT-CO1*	6698a	2.56 × 10^−5^	**0.0004**
D-loop	438c	0	0.0004	0.0548	*MT-CO3*	9499t	6.67 × 10^−5^	**0.0008**
*MT-ND6*	14628M	0.05	0.0012	0.1176	*MT-ND2*	4611a	0.0002	**0.0020**
*MT-ND5*	13237a	0.08	0.0022	0.1659	D-loop	438c	0.0004	**0.0027**
D-loop	302M	0.06	0.0033	0.1800	*MT-ND6*	14628M	0.0005	**0.0027**
*MT-ND2*	5251t	0.06	0.0033	0.1800	*MT-RNR1*	750G	0.0012	**0.0052**
*MT-TG*	10053a	0	0.0083	0.3152	*MT-ND5*	13237a	0.0013	**0.0052**
*MT-CYB*	15682a	0	0.0083	0.3152	*MT-ND2*	5251t	0.0014	**0.0052**
*MT-CYB*	15787t	0.07	0.0084	0.3152	D-loop	302M	0.0015	**0.0052**
D-loop	309.1C	0.14	0.0095	0.3266	*MT-CYB*	15787t	0.0028	**0.0089**
*MT-ND1*	3571c	0.18	0.0407	1	*MT-ND2*	4769G	0.0041	**0.0120**
*MT-ND2*	4611a	0.18	0.0407	1	*MT-RNR1*	1438G	0.0070	**0.0187**
*MT-ND6*	14429c	0	0.0471	1	D-loop	16380c	0.0143	**0.0345**
*MT-ATP6*	8695a	0	0.0471	1	*MT-ND1*	3571c	0.0148	**0.0345**
**PCOS + HT vs. HT**								
*MT-ND5*	13237a	0.12	0.0138	1	*MT-ND5*	13237a	0.0059	0.1655
					D-loop	309c	0.0350	0.4904
**PCOS + HT vs. C**								
*MT-CO1*	6698a	0	2.67 × 10^−6^	**0.0010**	*MT-CO1*	6698a	1.28 × 10^−5^	**0.0004**
*MT-CO3*	9499t	0	0.0003	0.0641	*MT-ND2*	4769G	8.77 × 10^−5^	**0.0014**
*MT-ND5*	13237a	0.07	0.0005	0.0641	*MT-ND5*	13237a	0.0004	**0.0036**
*MT-ND2*	5251t	0.05	0.0008	0.0734	*MT-ND2*	5251t	0.0006	**0.0036**
*MT-ND4*	11038a	6.7	0.0052	0.3980	*MT-CO3*	9499t	0.0007	**0.0036**
D-loop	438c	0	0.0111	0.7020	*MT-ND4*	11038del	0.0007	**0.0036**
*MT-ND4*	11038del	0.19	0.0138	0.7533	*MT-ND2*	4611a	0.0020	**0.0088**
					*MT-ND4*	11038a	0.0103	**0.0398**
					*MT-ND1*	3571c	0.0261	0.0817
					*MT-TS1*	7452a	0.0263	0.0817
					*MT-ND4*	11431c	0.0452	0.1274

Variants with a significant frequency difference in both Fisher’s exact and Mann–Whitney U analyses are highlighted in grey. Bold value indicates statistically significant differences after *p*-value adjustment (*p_adj_* < 0.05). Estimate value < 1 denotes variant’s underrepresentation.

## Data Availability

A large portion of original data presented in the study is included as Supplementary Material in this manuscript. Due to the ethical restrictions and data protection regulations (possibility of identification), the raw whole genome sequencing (WGS) of mtDNA data will be made available by the authors upon reasonable request.

## Supplementary Materials

The following supporting information can be downloaded at: https://www.mdpi.com/article/10.3390/antiox12111983/s1, Table S1: Identified mtDNA haplogroups; Tables S2 and S3: Functional enrichment analyses; Table S4: Prediction of significant variants’ effect.
